# Usual walking Pace and risk of 28 cancers– results from the UK biobank

**DOI:** 10.1186/s12885-025-14258-x

**Published:** 2025-05-14

**Authors:** Michael J. Stein, Hansjörg Baurecht, Patricia Bohmann, Pietro Ferrari, Béatrice Fervers, Emma Fontvieille, Heinz Freisling, Christine M. Friedenreich, Marc J. Gunter, Laia Peruchet-Noray, Anja M. Sedlmeier, Andrea Weber, Michael F. Leitzmann, Julian Konzok

**Affiliations:** 1 Department of Epidemiology and Preventive Medicine, University of Regensburg, Franz-Josef-Strauß-Allee 11, 93053 Regensburg, Germany; 2 Nutrition and Metabolism Branch, International Agency for Research on Cancer (IARC/WHO), Lyon, France; 3 Department of Prevention Cancer Environment, Centre Léon Bérard, Lyon, France; 4 INSERM UMR1296 Radiation: Defense, Health, Environment, Lyon, France; 5 Department of Cancer Epidemiology and Prevention Research, Cancer Care Alberta, Alberta Health Services, Calgary, AB Canada; 6 Departments of Oncology and Community Health Sciences, Cumming School of Medicine, University of Calgary, Calgary, AB Canada; 7 Cancer Epidemiology and Prevention Research Unit, School of Public Health, Imperial College, London, UK; 8 Department of Clinical Sciences, Faculty of Medicine, University of Barcelona, Barcelona, Spain; 9 Center for Translational Oncology, University Hospital Regensburg, Regensburg, Germany; 10 Bavarian Cancer Research Center (BZKF), Regensburg, Germany

**Keywords:** Walking Pace, Cancer prevention, UK biobank

## Abstract

**Background:**

Usual walking pace represents a practical indicator of overall health. However, its association with cancer development remains unexplored. We investigated the relation between self-reported walking pace and cancer risk.

**Methods:**

Using baseline UK Biobank data from 2006 to 2010, excluding the first two years of follow-up to reduce reverse causation, we employed multivariable Cox regression to assess the association between walking pace (slow, steady average, brisk) and risk of 28 cancer types, accounting for overall physical activity and walking volume.

**Results:**

After a median follow-up of 10.9 years (interquartile range 10.1–11.8), 8.3% of 334,924 participants received a cancer diagnosis. Brisk compared to slow walking pace was associated with multivariable-adjusted lower risks of five cancers, including anal (hazard ratio 0.30; 95% confidence interval: 0.14–0.63), hepatocellular carcinoma (0.39; 0.23–0.66), small intestine (0.46; 0.24–0.87), thyroid (0.50; 0.29–0.86), and lung cancer (0.60; 0.51–0.70). Our findings were consistent across various sensitivity analyses, which assessed sex and age differences, residual confounding, and reverse causation.

**Conclusions:**

Self-reported walking pace was inversely associated with risk of five cancer types, even when accounting for overall physical activity and walking volume. Adopting a brisk walking pace may represent a pragmatic target for public health interventions to decrease cancer risk, particularly in circumstances where increases in walking volume or frequency prove impractical.

**Supplementary Information:**

The online version contains supplementary material available at 10.1186/s12885-025-14258-x.

## Background

Cancer is one of the leading causes of mortality worldwide, accounting for nearly 10 million deaths in 2020. Projections indicate that the global cancer burden is set to rise significantly, with an expected increase of 47% by 2040, underscoring the growing impact of this disease [1]. Physical activity, particularly the volume of walking, is recognized for its role in reducing the risk of various cancers [2, 3].

Walking is often performed incidentally as part of daily life, yet may carry substantial potency in mitigating poor health outcomes. As a fundamental human movement pattern, likely rooted in our evolutionary biology, walking represents a simple, innate, and accessible behavior [4] that may serve as an effective preventive measure against chronic diseases, including cancer. The association between walking pace and cancer risk, however, remains unclear. Walking pace might be more than a mere indicator of physical activity; it might also reflect physical fitness [5], which shows an inverse relation to cancer risk [6].

Faster walking pace may lower cancer risk by reducing chronic inflammation [7], improving insulin sensitivity [8], favorably altering the gut microbiome composition [9], enhancing gut motility [10], and maintaining telomere length [11]. Therefore, understanding the association between walking pace and cancer, particularly independent of overall physical activity, is of considerable importance. Only one prior study has investigated walking pace in relation to cancer, finding an inverse association with lung cancer and a positive one with prostate cancer [12].

The current study aimed to provide novel evidence on the associations between walking pace and a large set of cancer sites within the UK Biobank. In view of the cost-effectiveness and simplicity of measuring walking pace, and its broad accessibility, these findings may inform recommendations to enhance cancer prevention strategies.

## Methods

### Study population and data collection

The UK Biobank, a prospective cohort study, recruited over 500,000 UK participants aged 40 to 69 years between 2006 and 2010. It collected sociodemographic, lifestyle, and extensive phenotypic data. Ethics approval was obtained from the North-West Multi-Centre Research Ethics Committee. Participants provided written informed consent [13].

Participants with prevalent malignant cancer (except non-melanoma skin cancer, *N* = 36,584) at cohort inclusion, those with cancer within the first two years of follow-up, and those with missing covariate data (*N* = 111,675) were excluded, leaving 334,924 participants for analysis (Additional File 1: eFigure 1).

### Usual walking pace

Usual walking pace was assessed in the baseline questionnaire [14], asking participants to categorize their pace as slow [< 3 miles per hour (mph)], steady average (3–4 mph), or brisk (> 4 mph). Those reporting not being able to walk were excluded. A previous study [15] showed that self-classified walking pace correlates well with accelerometry-measured speeds (slow: 1.39 m/s; average: 1.42 m/s; brisk: 1.45 m/s; *p* < 0.0001).

### Cancer ascertainment and cohort follow-up

Participants’ vital status was ascertained through linkage with routine health care data and national death registries [16]. Follow-up commenced at baseline and ended at cancer diagnosis, complete follow-up (February 2020 for England/Wales, January 2021 for Scotland) [17], loss-to-follow-up, or death, whichever occurred first. We focused on first primary cancers, treating multiple same-day diagnoses (*N* = 133) as a single case randomly. To ensure stability and interpretability of risk estimates, only cancers with over 100 cases were analyzed (classification in Additional File 1: eTable 1), including detailed subtype analyses (liver, stomach, colon) where case numbers allowed.

### Covariates

Potential confounding covariables were determined using evidence-based directed acyclic graphs (DAGs), with details in Additional File 1: eTable 2 and eFigure 2. DAGs are used to visualize causal structures and to identify confounding variables while distinguishing them from intermediate variables or colliders [18]. Briefly, we accounted for sex, age, and study region, and further adjusted for height, body mass index, grip strength, MVPA volume, walking volume, smoking, alcohol use, socio-economic status, education, sedentary behavior, healthy diet score, and cardiometabolic diseases (cardiovascular disease, type-2 diabetes). For gender-specific cancers, adjustments included menopausal status, screening, oral contraceptive use, hormone replacement therapy, childbirth history, and age at menarche/hysterectomy; prostate cancer analyses considered prostate specific antigen test use.

### Statistical analysis

We first investigated walking pace by estimating correlations with physical activity, grip strength, and self-reported health. Next, we ran multivariable-adjusted Cox proportional hazards regression models with age as the underlying time metric [19] to determine hazard ratios (HR) and 95% confidence intervals (CIs) for associations between walking pace and cancer risk. We checked the proportional hazards assumption via Schoenfeld residuals and visual inspection. To mitigate potential reverse causation, we did not consider cancer cases that occurred within the first two years of follow-up.

In sensitivity analyses, we excluded cancer cases that occurred within the first five years of follow-up. Interaction tests were conducted to identify subpopulations for whom walking pace may be particularly relevant, to avoid missing associations in specific groups, and to potentially inform more targeted prevention strategies. Therefore, we assessed whether the associations between walking pace and cancer risk were modified by age, sex, moderate-to-vigorous physical activity (MVPA), walking volume, cardiometabolic disease status, overall health status, and adiposity. All tests for interaction were conducted using the Wald test. We assessed potential residual confounding by smoking intensity by limiting analyses to never smokers and adjusting for pack-years of smoking instead of smoking status. To further evaluate whether the association of walking pace with cancer risk is independent of overall health, we additionally adjusted the models for self-rated health and excluded participants with poor self-rated health. To disentangle the relationship between walking pace, physical activity, and fitness, we repeated the analysis without adjustments for MVPA, walking volume, grip strength, and sedentary behavior. We used mortality through intentional self-harm as a negative control outcome to investigate unmeasured and residual confounding. Lastly, we additionally used Benjamini-Hochberg correction to account for multiple comparisons [20].

Analyses were conducted using R 4.3.2 [21] and the rms package [22], adhering to STROBE (Strengthening the Reporting of Observational studies in Epidemiology) guidelines [23].

## Results

We assessed cancer risk in 334,924 participants (51.1% women). Participants had a median age of 57 years (interquartile range 49–63). Median follow-up time was 10.9 years (Table 1). Walking pace was weakly positively correlated with physical activity and muscular fitness (Spearman’s r_s_: walking volume: 0.10, moderate physical activity: 0.10, vigorous physical activity: 0.20, grip strength: 0.11. Linear regression results were comparable, with β coefficients of 6.3, 6.3, 7.9, and 5.3 MET-hours for brisk walking, moderate physical activity, vigorous activity, and grip strength, respectively). Walking pace was modestly positively associated with self-rated health (Cramer’s V = 0.30) (Additional File 1: eTable 3).

**Table 1:** Baseline characteristics of the UK biobank between 2006–2010 (*N* = 334,924)

	Slow pace	Steady average pace	Brisk pace
**N**	21,319	171,396	142,209
**Sex**			
Women	11,025 (51.7%)	87,619 (51.1%)	72,631 (51.1%)
Men	10,294 (48.3%)	83,777 (48.9%)	69,578 (48.9%)
**Age at entry (years)**	58.3 (7.6)	56.6 (8.1)	54.8 (8.1)
**Townsend Index of Deprivation**	-0.2 (3.5)	-1.4 (3.0)	-1.7 (2.9)
**Qualification level**			
College or university	4,930 (23.1%)	56,210 (32.8%)	61,694 (43.4%)
A/AS, NVQ/HND/HNC or equivalent, other professional qualifications	4,848 (22.7%)	40,827 (23.8%)	33,234 (23.4%)
O/GCSEs, CSEs or equivalent	5,461 (25.6%)	48,227 (28.1%)	34,845 (24.5%)
None of the above	6,080 (28.5%)	26,132 (15.2%)	12,436 (8.7%)
**Height (cm)**	167.0 (9.4)	168.8 (9.2)	170.1 (9.3)
**Body mass index (kg/m** ^**2**^ **)**	31.3 (6.6)	27.9 (4.6)	25.8 (3.7)
**Weekly physical activity**			
First tertile	12,758 (59.8%)	60,900 (35.5%)	38,704 (27.2%)
Second tertile	5,146 (24.1%)	58,110 (33.9%)	49,809 (35.0%)
Third tertile	3,415 (16.0%)	52,386 (30.6%)	53,696 (37.8%)
**Walking volume (METhr/wk)**	11.5 (15.3)	17.3 (18.1)	18.2 (17.9)
**Grip strength (kg)**	29.2 (11.5)	33.2 (11.3)	34.9 (11.2)
**Sedentary behavior (hr)**	5.5 (3.1)	4.7 (2.5)	4.1 (2.4)
**Diet score**	3.3 (1.4)	3.5 (1.4)	3.8 (1.3)
**Smoking status**			
Never	9,487 (44.5%)	93,465 (54.5%)	83,730 (58.9%)
Former	8,222 (38.6%)	60,531 (35.3%)	46,737 (32.9%)
Current	3,610 (16.9%)	17,400 (10.2%)	11,742 (8.3%)
**Alcohol drinking status**			
Never	1,668 (7.8%)	6,850 (4.0%)	3,914 (2.8%)
Former	1,755 (8.2%)	5,399 (3.2%)	3,530 (2.5%)
Current	17,896 (83.9%)	159,147 (92.9%)	134,765 (94.8%)
**Family history of cancer**			
No	16,391 (76.9%)	130,782 (76.3%)	108,494 (76.3%)
Yes	4,928 (23.1%)	40,614 (23.7%)	33,715 (23.7%)
**Cardiometabolic disease**			
No	14,623 (68.6%)	151,086 (88.2%)	132,960 (93.5%)
Yes	6,696 (31.4%)	20,310 (11.8%)	9,249 (6.5%)

mph: miles per hour; A: Advanced; AS: Advanced Subsidiary; NVQ: National Vocational Qualification; HND: Higher National Diploma; HNE: Higher National Education; O: Ordinary Levels; GCSE: General Certificate of Education; CSE: Certificate of Secondary Education; METhr/wk: Metabolic equivalent of task hours per week

### Walking pace and cancer risk

We identified 11,978 cancer cases in women and 15,867 in men, encompassing 28 cancer types (Additional File 1: eTable 4).

**Fig. 1:**
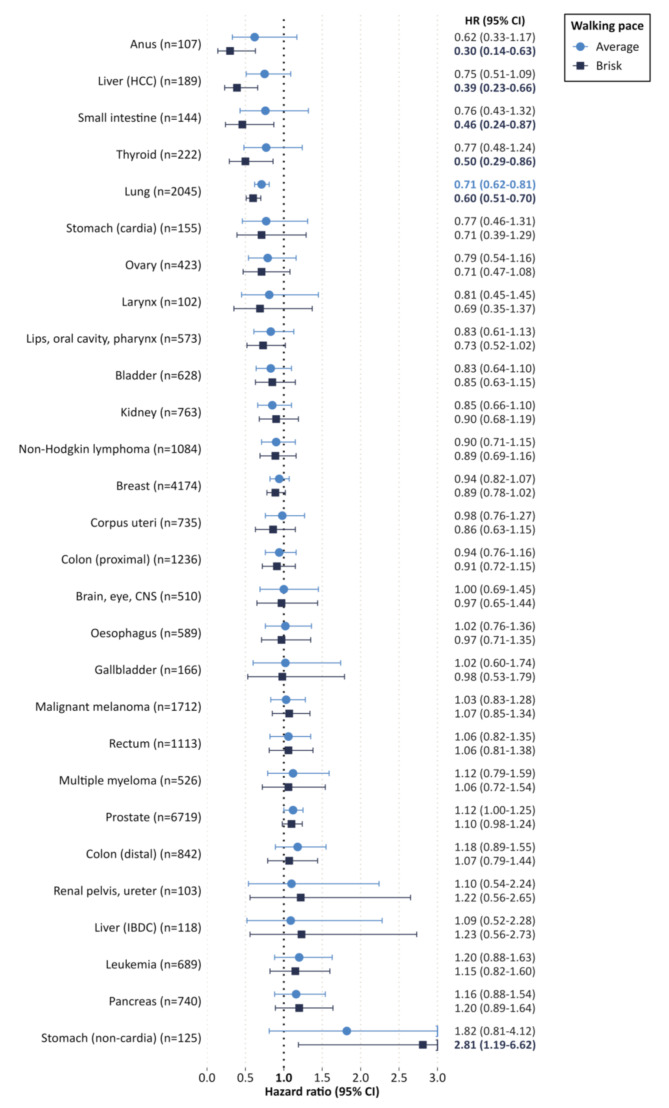
Hazard ratios (HR) and 95% confidence intervals (CI) for an average steady pace and brisk pace versus slow pace. HCC: Hepatocellular carcinoma; IBDC: Intrahepatic bile duct cancer; CNS: Central nervous system. Note: Proportional hazards assumption was violated for rectal cancer

Walking pace was inversely associated with five cancer types. Compared to a slow pace, a brisk pace was associated with lower risks of anal cancer (HR = 0.30; 95% CI: 0.14–0.63), hepatocellular carcinoma (0.39; 0.23–0.66), small intestine cancer (0.46; 0.24–0.87), thyroid cancer (0.50; 95% CI: 0.29–0.86), and lung cancer (0.60; 0.51–0.70). Decreased risks were observed for lip, oral cavity, and pharynx cancers combined and breast cancers, but these relations did not exhibit statistical significance. Conversely, brisk versus slow walking pace was positively associated with stomach (non-cardia) cancer (2.81; 1.19–6.62) and, albeit not statistically significantly, with prostate cancer (1.10; 0.98–1.24) (Fig. 1).

We found minimal interactions between walking pace, sex, age, MVPA, walking volume, cardiometabolic disease status, self-rated health, and adiposity with cancer risk (Additional File 1: eTable 5). Specifically, we noted a tendency for the inverse association between walking pace and anal cancer gradually weakening with higher MVPA levels (p for interaction = 0.327). Also, the inverse relation of walking pace to thyroid cancer gradually strengthened with higher levels of MVPA (p for interaction = 0.065) and walking volume (p for interaction = 0.023); and we observed an inverse association between walking pace and endometrial cancer among participants with the lowest levels of walking volume (p for interaction = 0.013) (Additional File 1: eTable 6). Walking pace was inversely associated with endometrial cancer among participants with overweight (p for interaction = 0.004) (Additional File 1: eTables 5 and 7). Furthermore, walking pace was inversely associated with proximal colon cancer only among those with cardiometabolic diseases (p for interaction = 0.014), and with small intestine cancer only among those without cardiometabolic diseases (p for interaction = 0.006) (Additional File 1: eTables 5 and 8).

Results were generally consistent by sex, except for oesophageal cancer (p for interaction = 0.004), for which an inverse association with walking pace was noted among women (Additional File 1: eFigures 4, 5). Lung cancer was consistently inversely associated with walking pace across age groups. Further comparisons were limited by reduced case numbers, but among those aged < 60 years, female-specific cancers tended to show inverse associations with walking pace (Additional File 1: eFigure 6).

### Additional and sensitivity analyses

After excluding the first five years of follow-up (10,131 exclusions), results remained consistent. Additionally, proximal colon cancer was inversely associated with walking pace (Additional File 1: eFigure 3). Among never smokers, findings were attenuated and became statistically non-significant, except for hepatocellular carcinoma (Additional File 1: eTable 9). Replacing smoking status with smoking dose did not alter the lung cancer results (Additional File 1: eTable 10). Adjusting for self-rated health and excluding participants with poor self-rated health slightly attenuated associations, but results remained materially unchanged (Additional File 1: eFigures 7 and 8). Our findings appeared stronger when not adjusting for MVPA, walking volume, grip strength, and sedentary behavior, with a borderline inverse association between walking pace and breast cancer (Additional File 1: eFigure 9). Walking pace was not associated with our negative control outcome (Additional File 1: eTable 11). After adjustment for multiple comparisons, only anal cancer (p-adjusted = 0.014), hepatocellular carcinoma (p-adjusted = 0.007), and lung cancer (p-adjusted < 0.001) remained statistically significant (Additional File 1: eTable 12).

## Discussion

In this large cohort of men and women, we examined the relations between walking pace and 28 different cancer types. Our analysis revealed that a brisk walking pace was associated with reduced risk of five cancers, including anal, liver, small intestine, thyroid, and lung cancers.

Our study indicates that self-reported walking pace may represent an important health determinant, extending previous research linking this factor with overall health and vitality [24, 25], mortality [12, 26–32], cardiovascular disease [33, 34], cardiometabolic traits [28, 35–38], and telomere length [11]. We linked walking pace to certain cancer sites not currently included in international physical activity guidelines [39].

Increased walking pace may be linked to lower cancer risk through decreased chronic inflammation and insulin resistance pathways. Prior studies found that walking pace is inversely related to inflammatory markers like C-reactive protein, interleukin 6, and tumor necrosis factor alpha [7]. Walking pace also improves insulin sensitivity and reduces insulin resistance [8]. Maintaining telomere length, crucial in many cancers [40], may be a further mechanism impacted by walking pace, as brisk walkers exhibit longer telomeres, indicative of better health [11].

We found a decreased risk of lung cancer with elevated walking pace. This aligns with previous observational research linking brisk walking pace to reduced lung cancer risk in men [12] and with data showing that improved cardiovascular fitness, potentially akin to brisk walking [41], decreases lung cancer risk [42]. Our results also echo findings demonstrating inverse relations of genetically-predicted walking pace to lung cancer [28]. Lung cancer is strongly influenced by smoking but also by cardiorespiratory fitness [43]. Brisk walking may enhance aerobic capacity in individuals with impaired pulmonary function [44]. Therefore, faster walking pace may protect against lung cancer development, possibly through improved tissue oxygenation and lower chronic inflammation. Although smoking is a major potential confounder in studies of lung cancer risk [45], our analysis, robustly adjusted for smoking, showed minimal residual confounding. Lung cancer results remained materially unchanged after adjusting for smoking intensity instead of smoking status. While the association appeared weaker in never smokers, possibly due to reduced statistical power (only 301 lung cancer cases), the directionality of the relation persisted.

A brisk walking pace was associated with a lower risk of liver cancer, consistent with previous research in U.S. adults [46], even after accounting for physical activity. Brisk walking is associated with improved insulin sensitivity [8], which lowers the risk for non-alcoholic fatty liver disease and its progression to hepatocellular carcinoma [47]. This may be particularly relevant given the strong metabolic underpinnings of liver cancer.

Brisk walking was associated with decreased risk of anal cancer. Anal cancer is etiologically linked to immunological and inflammatory pathways, particularly in the context of HPV infection [48]. Brisk walking decreases systemic chronic inflammation [7], which may enhance immune surveillance and thereby help mitigate oncogenic viral persistence or progression in susceptible tissues. Although not statistically significantly associated in our main analysis, we observed an inverse association between walking pace and proximal colon cancer in participants with cardiometabolic diseases. This is consistent with the established link between physical activity and colon cancer [45], including low-intensity activities like walking [49] and cardiorespiratory fitness showing benefits [50].

Moreover, we found an inverse association between walking pace and thyroid cancer. The relation between walking and thyroid cancer is under-researched, though some evidence suggests an inverse association between daily walking volume and thyroid cancer [51]. Metabolic health and insulin resistance may play a role in thyroid carcinogenesis [52]. Faster walking pace is related to better metabolic profiles and lower adiposity [53], which may reduce thyroid cancer risk.

We observed an inverse association between walking pace and small intestine cancer. Although rare and understudied, cancers of the small intestine may be influenced by gut health [54]. In addition to reducing systemic inflammation [7], a faster walking pace may decrease small intestine cancer risk by improving gut motility [10] and microbial composition [9], potentially reducing contact time with carcinogens and promoting mucosal integrity. These results warrant cautious interpretation due to limited existing literature.

Walking pace was positively associated with non-cardia stomach cancer. However, this association is likely non-causal and could reflect residual confounding or reverse causality. Individuals who walk briskly may be more health-conscious and undergo more frequent medical checkups, increasing the chance of incidental diagnosis and detection bias. Alternatively, non-cardia gastric cancer is often linked to Helicobacter pylori infection and other non-metabolic factors [55] that are less influenced by physical activity. The wide CIs and lack of consistency across sensitivity analyses suggest this finding should be interpreted cautiously.

In addition, we found a statistically non-significant positive relation to prostate cancer risk with higher walking pace, supported by one previous observational UK Biobank study [12] and cardiorespiratory fitness research [56, 57]. The underlying mechanisms are unclear, but health-conscious men who often walk faster, may undergo more cancer screening, leading to higher prostate cancer detection rates in fit men, rather than indicating a direct causal association [56].

While we found inverse associations for several cancers previously linked to physical activity [58], such as colorectal, lung, and liver cancers, associations with others, including breast, endometrial, or esophageal cancers, were only observed in sensitivity analyses. This may reflect the fact that walking pace is not solely a measure of physical activity in our classical understanding, defined by frequency, intensity, and duration, but rather an integrative behavioral phenotype that combines aspects of physical activity, fitness, and perceived health. Consistent with this, we found associations with additional cancer types, suggesting an impact of walking pace on cancer risk beyond physical activity alone. Future research is warranted to investigate this expanded list of cancers. We adjusted for physical activity volume, which may have accounted for its pathway-specific influence on cancer risk. Walking pace, as an extension of the traditional concept of physical activity, may be associated with cancer through alternative mechanisms, which remain to be elucidated. Beyond mechanisms related to the immune system, insulin sensitivity, gut motility, and the gut microbiome, walking pace may act through increased fitness, as higher walking pace is typically achieved through improved muscular balance, strength, and performance and is related to higher energetic efficiency [59]. However, the current state of evidence is limited, and further studies are needed to uncover the biologic mechanisms underlying brisk walking to disentangle the walking pace-physical activity-fitness relations and their associations with cancer. In addition, future studies should acknowledge the compositional nature of human movement by accounting for time spent on physical activity at different intensities, time spent walking at different velocities, time spent sedentary, and time spent sleeping. Some of the cancers we studied are rare, such as anal or oral cancers, while others, like colorectal and lung cancers, are common. Therefore, walking pace could become a relevant public health target as it may play a crucial role in supporting cancer prevention given its simplicity as a clinical assessment and intervention target.

### Strengths and limitations

The primary strength of our study is its novel exploration of the associations between walking pace and previously unexamined cancer sites. We analyzed a large cohort of both men and women, ensuring substantial case numbers and statistical power. The validity of our outcome data was bolstered by linkage to hospital inpatient data, rather than relying on self-reported disease status.

The following limitations should be mentioned. We relied on self-reported walking pace, which, despite previous studies showing strong correlations with measured pace [15], may not accurately reflect participants’ actual walking speed. Small case numbers for some cancers led to wide CIs. Despite comprehensive model adjustments and extensive sensitivity analyses, residual or unmeasured confounding cannot be entirely ruled out, particularly for factors not fully captured in the dataset, such as undiagnosed or subclinical comorbidities, healthcare-seeking behavior, or early-life exposures. Furthermore, using grip strength as a proxy for physical fitness may have introduced residual confounding by muscular fitness. Also, assessing participants’ cardiorespiratory fitness would have provided further insights to disentangle the interplay between walking pace and fitness. Finally, the study’s focus on European ancestry participants potentially limits its generalizability.

## Conclusions

We found inverse associations between walking pace and the risk of five cancers, including anal, liver, small intestine, thyroid, and lung cancers. These findings underscore self-reported walking pace as a valuable health indicator, highlighting potential implications for medical practice, public health, and research. Self-reported walking pace represents a simple yet informative metric that may play a crucial role in supporting cancer prevention efforts.

## Electronic supplementary material

Below is the link to the electronic supplementary material.

Supplementary Material 1

## Data Availability

UK Biobank is an open access resource. Bona fide researchers can apply to use the UK Biobank dataset by registering and applying at http://ukbiobank.ac.uk/register-apply/.

## Abbreviations

CI: Confidence interval
DAG: Directed acyclic graphs
HR: Hazard ratio
mph: Miles per hour
MVPA: Moderate-to-vigorous physical activity
STROBE: Strengthening the Reporting of Observational studies in Epidemiology
