# Supplementary material for: Usual walking Pace and risk of 28 cancers– results from the UK biobank
Source: BMC Cancer. 2025 May 14;25:869. doi: 10.1186/s12885-025-14258-x (PMC12077053; doi:10.1186/s12885-025-14258-x)

**Additional file 1: Supplementary material**

**Usual walking pace and risk of 28 cancers – results from the UK Biobank**

**Corresponding author** Michael J. Stein, Department of Epidemiology and Preventive Medicine, University of Regensburg, Franz-Josef-Strauß-Allee 11, 93053 Regensburg, Germany, Email: michael.stein@ukr.de

[Supplementary Table 1. International Classification of Diseases (ICD-10) classification of cancer subtypes](#_Toc194683622)

[Supplementary Table 2. Covariate details](#_Toc194683623)

[Supplementary Table 3. Associations between walking pace and physical activity, grip strength, and self-rated health](#_Toc194683624)

[Supplementary Table 4. Cancer cases by gender](#_Toc194683625)

[Supplementary Table 5. P for interaction between walking pace and sex, age, moderate-to-vigorous physical activity, walking volume, cardiometabolic disease status, health status, adiposity, and smoking status](#_Toc194683626)

[Supplementary Table 6. Cox model results across levels of moderate-to-vigorous physical activity and walking volume](#_Toc194683627)

[Supplementary Table 7. Cox model results by body mass index](#_Toc194683628)

[Supplementary Table 8. Cox model results by cardiometabolic disease status](#_Toc194683629)

[Supplementary Table 9. Cox model results for never smokers](#_Toc194683630)

[Supplementary Table 10. Lung cancer risk adjusted for pack years of smoking](#_Toc194683631)

[Supplementary Table 11. Negative control outcome](#_Toc194683632)

[Supplementary Table 12. P-values after correction for multiple comparisons](#_Toc194683633)

[Supplementary Figure 1. Flow chart of participant inclusion and exclusion](#_Toc194683634)

[Supplementary Figure 2. Directed acyclic graph](#_Toc194683635)

[Supplementary Figure 3. Cox model results after exclusion of first five years of follow-up](#_Toc194683636)

[Supplementary Figure 4. Cancer risk among men](#_Toc194683637)

[Supplementary Figure 5. Cancer risk among women](#_Toc194683638)

[Supplementary Figure 6. Cancer risk by age groups](#_Toc194683639)

[Supplementary Figure 7. Cancer risk after adjustment for overall health status](#_Toc194683640)

[Supplementary Figure 8. Cancer risk after exclusion of participants with poor self-rated health](#_Toc194683641)

[Supplementary Figure 9. Cancer risk without adjustment for moderate-to-vigorous physical activity, walking volume, grip strength, and sedentary behavior](#_Toc194683642)

# International Classification of Diseases (ICD-10) classification of cancer subtypes

| **Cancer** | **ICD-10 code** |
| --- | --- |
| Lips, oral cavity, pharynx | C00–C14 |
| Oesophagus | C15 |
| Stomach (cardia) | C16.0 |
| Stomach (non-cardia) | C16.1–C16.6 |
| Small intestine | C17.0 |
| Rectum | C19–C20 |
| Colon (proximal) | C18.0–C18.5 |
| Colon (distal) | C18.6–C18.7 |
| Anus | C21 |
| Liver (HCC) | C22.0 |
| Liver (IBDC) | C22.1 |
| Gallbladder | C23–C24 |
| Pancreas | C25 |
| Lung | C34 |
| Malignant melanoma | C43 |
| Breast | C50 |
| Corpus uteri | C54 |
| Ovary | C56 |
| Prostate | C61 |
| Kidney | C64 |
| Bladder | C67 |
| Brain, CNS, eye | C69–C72 |
| Thyroid | C73 |
| Non-Hodgkin lymphoma | C82–C85 |
| Multiple myeloma | C90 |
| Leukemia | C91–C95 |
| HCC: Hepatocellular carcinoma; IBDC: Intrahepatic bile duct cancer; CNS: Central nervous system.  Note: Breast cancer was only considered among women. | |

# Covariate details

| **Information on confounding variables.** | | | |
| --- | --- | --- | --- |
| **Covariate** | **UK Biobank – variable identification number** | **Calculation** | **Further Comments** |
| Age at menarch | ID 2714: Age when periods started (menarche) |  | Rejected when:  < 5 and > Participants age or > 25  Asked to confirm when:  < 6 and > 20  “Prefer not to answer” and “Do not know” were coded NA |
| Age group | ID 21022: Age at recruitment | Categorized to groups with 5-year steps:  35-39, 40-44, 45-49, 50-54, 55-59, 60-64, 65-69, 70-75 | “Prefer not to answer” were coded NA |
| Alcohol use status | ID 20117: Alcohol drinker status | Self-reported alcohol drinker status: never, previous, current |  |
| Body mass index | ID 21001: Body mass index (BMI) | Weight (kg) divided by height (m) squared | Value not present if either weight or height were not available |
| Cardiometabolic diseases | ID 2443: Diabetes diagnosed by doctor  ID 6150: Vascular/heart problems diagnosed by doctor  ID 41202: Diagnoses – main ICD10  ID 41203: Diagnoses – main ICD9  ID 41204: Diagnoses – secondary ICD10  ID 41205: Diagnoses – secondary ICD9  ID 41262: Date of first in-patient diagnosis – main ICD10  ID 41263: Date of first in-patient diagnosis – main ICD9  ID 41270: Diagnoses – ICD10  ID 41271: Diagnoses – ICD9  ID 41280: Date of first in-patient diagnosis – ICD10  ID 41281: Date of first in-patient diagnosis – ICD9 | **Prevalent cardiovascular diseases:**  The following baseline self-reported diseases were used:   - 1 = Heart attack - 2 = Angina - 3 = Stroke   The following diseases and their ICD codes were defined as cardiovascular diseases:   - Angina pectoris: ICD 10: I20.0 - I20.9 & ICD9: 4139 - Acute myocardial infarction: ICD 10: I21.0 – I21.9 & ICD9: 4109 - Other acute ischemic heart diseases: ICD10: I24.0 – I24.9 & ICD9: 4119 - Chronic ischemic heart diseases: ICD10: I25.0 – I25.9 & ICD9: 4140, 4148, 4149 - Atrial fibrillation: ICD10: I48.0-I48.9 & ICD9: 4273 - Other cardiac arrhythmias: ICD10: I49.0 – I 49.9 & ICD9: 4270-4279 - Heart failure: ICD10: I50.0 – I50.9 & ICD9: 4280, 4281 - Cerebrovascular diseases (incl. stroke): ICD10: I60.0 – I60.9; I61.0 – I61.9; I62.0 – I62.9; I63.0 – I63.9; I64; I65.0 – I65.9; I66.0 – I66.9; I67.0 – I67.9; I68.0 – I68.9; I69.0 – I69.9 & ICD9: 4309, 4319, 4321, 4331, 4339, 4349, 4359, 4369, 4373, 4379, 4389 - Atherosclerosis: ICD10: I70.0 – I70.9 & ICD9: 4400, 4401, 4408, 4409 - Other peripheral vascular diseases: ICD10: I73.0 – I73.9 & ICD9: 4430, 4439   **Prevalent type 2 diabetes:**  Baseline self-reported diagnosis of diabetes was used as well as the following ICD codes:   - ICD10: E11.0 – E11.9   ICD9: 25000 |  |
| Child birth history | ID 2734: Number of live births | Self-reported number of births | Below 0 and above 25 was rejected, above 12 was asked to confirm |
| Diet | ID 1289: Cooked vegetable intake  ID 1299: Salad/raw vegetable intake  ID 1309: Fresh fruit intake  ID 1319: Dried fruit intake  ID 1329: Oily fish intake  ID 1339: Non-oily fish intake  ID 1349: Processed meat intake  ID 1359: Poultry intake  ID 1369: Beef intake  ID 1379: Lamb/Mutton intake  ID 1389: Pork intake  ID 1438: Bread intake  ID 1448: Bread type  ID 1458: Cereal intake  ID 1468: Cereal type | Building a healthy diet score based on Lourida et al. (2) ranging from 0 – 7 by giving one point per fulfilled nutritional category:   - Fruits: 3 servings/day - Vegetables: 3 servings/day - Fish: 2 servings/week - Processed meats: 1 serving/week - Unprocessed red meat: 1.5 servings/week - Whole grains: 3 servings/day - Refined grains: 1.5 servings/day | No inclusion of dairy intake (ID 1408, ID 1418, ID 1428)  Corrections:   - Vegetables & Fruit: <0 = 0; >5=5 |
| Education | ID 6138: Qualifications | Categorization in four groups:  1 = University or College degree  2 = A-level/professional qualification/HNC/NVQ  3 = 0-levels/CSE  4 = None | “Prefer not to answer” was coded NA |
| Family history of cancer | ID 20107: Illnesses of father  ID 20110: Illnesses of mother | Family history of cancer was yes, when any was true  3 = Lung cancer  4 = Bowel cancer  5 = Breast cancer  13 = Prostate cancer |  |
| Grip strength | ID 46: Hand grip strength (left)  ID 47: Hand grip strength (right) | The maximum was considered as maximal hand grip strength |  |
| Height | ID 50: Standing height | Measured in cm |  |
| History of prostate cancer screening | ID 2365: Ever had prostate specific antigen (PSA) test | Self-reported use of blood test for prostate cancer | “Do not know” and “prefer not to answer” were coded NA |
| History of breast cancer screening | ID 2674: Ever had breast cancer screening / mammogram | Self-reported breast cancer screening: yes, no | “Do not know” and “prefer not to answer” were coded NA |
| History of hysterectomy | ID 2824: Age at hysterectomy  ID 2724: Had menopause | History of hysterectomy was categorized (yes, no) when participant indicated a previous hysterectomy | Age rejected when:  < 0, > Participants age  Age asked to confirm when:  < 35, > 69, < Age when had the only child or < Age when had the last child  “Prefer not to answer” and “Do not know” were coded NA |
| Hormone replacement therapy | ID 2814: Ever used hormone-replacement therapy (HRT) | Baseline touchscreen question: “Have you ever used hormone replacement therapy (HRT)?” | “Do not know” and “prefer not to answer” were coded NA |
| Menopausal status | ID 2724: Had menopause | Self-reported menopausal status: yes, no | „Not sure - had a hysterectomy“, “not sure – other reason“, and “prefer not to answer” were coded NA |
| Oral contraceptive use | ID 2784: Ever taken oral contraceptive pill |  | “Do not know” and “prefer not to answer” were coded NA |
| Sedentary behavior | ID 1070: Time spent watching TV  ID 1080: Time spent using computer  ID 1090: Time spent driving | Total sedentary behavior was calculated by adding up time spent watching television, time spent using computer and time spent driving.  Sedentary behavior while working was not included in our calculation. | The data was corrected by a truncation to 24h, if the sum was >24h.  Whenever there was a missing in either variable, the total sedentary behavior was considered as missing (n = 19,038) |
| Self-reported physical activity | ID 864: Number of days/week walked 10+ minutes  ID 874: Duration of walks  ID 884: Number of days/week of moderate physical activity 10+ minutes  ID 894: Duration of moderate activity  ID 904: Number of days/week of vigorous physical activity 10+ minutes  ID 914: Duration of vigorous activity | Categorization based on age- and sex-standardized tertiles of total physical activity per week based on:  Various continuous scores:   - Walking MET-min/week =   3.3 * walking minutes * walking days   - Moderate MET-min/week =   4 * moderate minutes * moderate days   - Vigorous MET-min/week = 8 * vigorous minutes * vigorous days   Total score:  MET-min/week = Walking + Moderate + Vigorous MET-min/week scores | The data were corrected by truncation of high values (>180 was set to 180 for each walking duration, vigorous and moderate physical activity).  Outliers were defined as individuals with a sum of walking, moderate and vigorous physical activity of above 960 minutes per day.  Responses below 10 minutes were set to 0.  Whenever there was a missing variable, the total physical activity was considered missing (n = 118,333)  These corrections were based on UK Biobank IPAQ Guidelines (3). |
| Smoking status | ID 20116: Smoking status | Self-reported smoking status: never, previous, current | “Prefer not to answer” were coded NA |
| Socio-economic status | ID 22189: Townsend deprivation index at recruitment | A composite score of employment, ownership of car and home, household overcrowding and postcode; higher values indicate a higher degree of deprivation. | The calculation happens prior to participating.  The values are rounded to 2 decimal places. |
| Study region | ID 54: UK Biobank Assessment centre | The 22 single centers were grouped according to their country of origin. |  |
| Walking volume | ID 864: Number of days/week walked 10+ minutes  ID 874: Duration of walks  ID 884: Number of days/week of moderate physical activity 10+ minutes | Walking MET-min/week =  3.3 * walking minutes * walking days | Same corrections as for total physical activity. |
| CSE: Certificate of Secondary Education; HNC: Higher National Certificate; MET: Metabolic equivalent of task; NVQ: National Vocational Qualification  [1] Bradbury KE, Murphy N, Key TJ. Diet and colorectal cancer in UK Biobank: a prospective study. International Journal of Epidemiology. 2019;49(1):246-58. doi:10.1093/ije/dyz064  [2] Lourida I, Hannon E, Littlejohns TJ, et al. Association of Lifestyle and Genetic Risk With Incidence of Dementia. JAMA. 2019;322(5):430-7. doi:10.1001/jama.2019.9879  [3] UK Biobank. Guidelines for data processing and analysis of IPAQ. Available at: https://biobank.ctsu.ox.ac.uk/crystal/refer.cgi?id=540 | | | |

# Associations between walking pace and physical activity, grip strength, and self-rated health

| **Correlation between walking pace and physical activity and grip strength** | | |
| --- | --- | --- |
| Correlation with | Spearman *r_s_* | Regression coefficient |
| Weekly walking | 0.10 | 5.46 (average steady) / 6.26 (brisk) |
| Weekly moderate physical activity | 0.10 | 5.13 (average steady) / 6.25 (brisk) |
| Weekly vigorous physical activity | 0.20 | 4.88 (average steady) / 7.90 (brisk) |
| Maximum grip strength | 0.11 | 3.87 (average steady) / 5.30 (brisk) |
| Physical activity as measured in metabolic equivalent of task (MET)-hours; grip strength as measured in kilograms.  The regression models were adjusted for walking pace, sex, age, study center, and body mass index. | | |

| **Number of participants per total weekly physical activity tercile and walking pace** | | | |
| --- | --- | --- | --- |
|  | Slow pace | Average steady pace | Brisk pace |
| Lowest | 12,758 (60%) | 60,900 (36%) | 38,704 (27%) |
| Medium | 5,146 (24%) | 58,110 (34%) | 49,809 (35%) |
| Highest | 3,415 (16%) | 52,386 (31%) | 53,696 (38%) |
| Physical activity measured by International Physical Activity Questionnaire (IPAQ)  Pearson’s Chi-squared test for physical activity group (low, moderate, high) and walking pace:  *X^2^* = 10114  p-value < 2.2*10^-16^  Cramer’s V = 0.1229 | | | |

| **Number of participants per self-reported overall health and walking pace** | | | |
| --- | --- | --- | --- |
|  | Slow pace | Average steady pace | Brisk pace |
| Poor | 5,604 (26%) | 4,452 (3%) | 1,314 (1%) |
| Fair | 8,799 (42%) | 37,936 (22%) | 15,861 (11%) |
| Good | 6,160 (29%) | 105,224 (62%) | 86,175 (61%) |
| Excellent | 611 (3%) | 23,391 (14%) | 38,696 (27%) |
| Pearson’s Chi-squared test for self-reported overall health and walking pace:  *X^2^* = 61413  p-value < 2.2*10^-16^  Cramer’s V = 0.3031 | | | |

# Cancer cases by gender

| **Cancer** | **Women**  **N=11,978** | **Men**  **N=15,867** |
| --- | --- | --- |
| Anus | 70 | 37 |
| Bladder | 123 | 505 |
| Brain, eye, CNS | 193 | 317 |
| Breast | 5,008 | 0 |
| Colon (distal) | 334 | 508 |
| Colon (proximal) | 574 | 662 |
| Corpus uteri | 794 | 0 |
| Gallbladder | 77 | 89 |
| Kidney | 241 | 522 |
| Larynx | 7 | 95 |
| Leukemia | 251 | 438 |
| Lips, oral cavity, pharynx | 185 | 388 |
| Liver (HCC) | 33 | 156 |
| Liver (IBDC) | 62 | 56 |
| Lung | 931 | 1,114 |
| Malignant melanoma | 782 | 930 |
| Multiple myeloma | 215 | 311 |
| Non-Hodgkin lymphoma | 449 | 635 |
| Oesophagus | 146 | 443 |
| Ovary | 487 | 0 |
| Pancreas | 318 | 422 |
| Prostate | 0 | 7,037 |
| Rectum | 379 | 734 |
| Renal pelvis, ureter | 33 | 70 |
| Small intestine | 61 | 83 |
| Stomach (cardia) | 29 | 126 |
| Stomach (non-cardia) | 47 | 78 |
| Thyroid | 149 | 73 |
| HCC: Hepatocellular carcinoma; IBDC: Intrahepatic bile duct cancer; CNS: Central nervous system | | |

# P for interaction between walking pace and sex, age, moderate-to-vigorous physical activity, walking volume, cardiometabolic disease status, health status, adiposity, and smoking status

| **Cancer** | **Sex** | **Age group** | **MVPA** | **Walking** | **CMD** | **BMI** |
| --- | --- | --- | --- | --- | --- | --- |
| Anus | 0.8073 | 0.9271 | 0.3267 | 0.9770 | 0.9507 | 0.9840 |
| Bladder | 0.2987 | 0.9954 | 0.8430 | 0.9559 | 0.7747 | 0.2591 |
| Brain, eye, CNS | 0.9557 | 0.2526 | 0.6506 | 0.7793 | 0.6917 | 0.4732 |
| Breast | - | 0.6168 | 0.5166 | 0.3538 | 0.7565 | 0.4282 |
| Colon (distal) | 0.1991 | 0.5416 | 0.9227 | 0.4936 | 0.4698 | 0.2205 |
| Colon (proximal) | 0.5375 | 0.7026 | 0.5446 | 0.8615 | **0.0139** | 0.6175 |
| Corpus uteri | - | 0.2480 | 0.3417 | **0.0131** | 0.5909 | **0.0044** |
| Gallbladder | 0.7814 | 0.9842 | 0.2914 | 0.1156 | 0.6877 | 0.1164 |
| Kidney | 0.5143 | 0.6431 | 0.8870 | 0.3464 | 0.9373 | 0.6669 |
| Larynx | **0.0386** | 0.9240 | 0.6435 | 0.4436 | 0.5645 | 0.6404 |
| Leukemia | 0.3050 | 0.6193 | 0.4516 | 0.3882 | 0.3114 | 0.3645 |
| Lips, oral cavity, pharynx | 0.9527 | 0.7885 | 0.4962 | 0.3659 | 0.7458 | 0.4267 |
| Liver (HCC) | 0.9137 | 0.9786 | 0.4970 | 0.5950 | 0.4938 | 0.9683 |
| Liver (IBDC) | 0.0857 | 0.9498 | 0.5001 | 0.4535 | 0.9483 | 0.5620 |
| Lung | 0.0770 | 0.6180 | 0.4801 | 0.8237 | 0.6997 | 0.7189 |
| Malignant melanoma | 0.1253 | 0.7996 | 0.4215 | 0.6583 | 0.6182 | 0.7544 |
| Multiple myeloma | 0.3741 | 0.5079 | 0.1191 | 0.9894 | 0.9128 | 0.2556 |
| Non-Hodgkin lymphoma | 0.1220 | 0.9963 | 0.9299 | 0.7858 | 0.3022 | 0.4229 |
| Oesophagus | **0.0036** | 0.7346 | 0.3869 | 0.3086 | 0.7198 | 0.5057 |
| Ovary |  | 0.9334 | 0.4953 | 0.3332 | 0.6410 | 0.9924 |
| Pancreas | 0.3006 | 0.9513 | 0.8457 | 0.8721 | 0.9689 | 0.3168 |
| Prostate | - | 0.1663 | 0.6015 | 0.8933 | 0.0902 | 0.3110 |
| Rectum | 0.0806 | 0.2217 | 0.8734 | 0.1745 | 0.7955 | 0.7017 |
| Renal pelvis, ureter | 0.1333 | 0.9986 | 0.6625 | 0.6884 | 0.8243 | 0.4656 |
| Small intestine | 0.6548 | 0.2025 | 0.2951 | 0.1919 | **0.0058** | 0.4028 |
| Stomach (cardia) | 0.4928 | 0.9618 | 0.8603 | 0.6626 | 0.4504 | 0.7937 |
| Stomach (non-cardia) | 0.2805 | 0.4148 | 0.9705 | 0.6441 | 0.1989 | 0.9296 |
| Thyroid | 0.8504 | 0.8306 | 0.0648 | **0.0233** | 0.4135 | 0.4515 |
| BMI: Body mass index; HCC: Hepatocellular carcinoma; IBDC: Intrahepatic bile duct cancer; CMD: cardiometabolic disease; CNS: Central nervous system; MVPA: moderate-to-vigorous physical activity.  *Proportional hazard assumption was violated for prostate cancer. | | | | | | |

# Cox model results across levels of moderate-to-vigorous physical activity and walking volume

|  | **Low MVPA** | **Median MVPA** | **High MVPA** |
| --- | --- | --- | --- |
|  | Hazard ratio (95% CI) | | |
| Anus | 0.22 (0.10-0.50) | 0.34 (0.14-0.84) | 0.68 (0.12-3.67) |
| Liver (HCC) | 0.34 (0.19-0.61) | 0.36 (0.21-0.61) | 0.39 (0.22-0.71) |
| Small intestine | 0.40 (0.19-0.83) | 0.42 (0.22-0.81) | 0.45 (0.22-0.91) |
| Thyroid | 0.67 (0.37-1.21) | 0.55 (0.32-0.93) | 0.38 (0.21-0.70) |
| Lung | 0.58 (0.49-0.69) | 0.58 (0.50-0.68) | 0.59 (0.49-0.70) |
| Stomach (non-cardia) | 2.92 (1.17-7.31) | 2.96 (1.25-7.03) | 3.04 (0.98-9.42) |
|  |  |  |  |
|  | **Low walking** | **Median walking** | **High walking** |
| Anus | 0.30 (0.13-0.68) | 0.30 (0.14-0.63) | 0.29 (0.13-0.68) |
| Liver (HCC) | 0.37 (0.20-0.67) | 0.37 (0.22-0.64) | 0.37 (0.21-0.67) |
| Small intestine | 0.35 (0.17-0.73) | 0.45 (0.23-0.91) | 0.70 (0.27-1.83) |
| Thyroid | 0.71 (0.39-1.32) | 0.56 (0.32-0.97) | 0.37 (0.21-0.65) |
| Lung | 0.57 (0.48-0.68) | 0.59 (0.50-0.69) | 0.63 (0.53-0.75) |
| Stomach (non-cardia) | 3.18 (1.21-8.37) | 2.85 (1.19-6.79) | 2.34 (0.94-5.84) |
| Corpus uteri | 0.69 (0.50-0.96) | 0.83 (0.61-1.13) | 1.13 (0.74-1.73) |
| CI: Confidence interval; HCC: Hepatocellular carcinoma; MVPA: Moderate-to vigorous-physical activity.  Results given for brisk walking pace compared to slow pace. Moderate-to-vigorous-physical activity (MVPA) as measured in metabolic equivalent of task (MET)-hours. Low MVPA = 4 METhrs; Median MVPA = 16 METhrs; High MVPA = 36 METhrs.  Walking volume as measured in MET-hours. Low walking = 5.50 METhrs; Median walking = 11.55 METhrs; High walking = 23.10 METhrs. | | | |

# Cox model results by body mass index

|  | **BMI < 25 kg/m^2^** | **BMI 25-29.9 kg/m^2^** | **BMI >= 30 kg/m^2^** |
| --- | --- | --- | --- |
|  | Hazard ratio (95% CI) | Hazard ratio (95% CI) |  |
| Corpus uteri | 3.79 (0.53-27.24) | 0.36 (0.23-0.56) | 0.65 (0.38-1.11) |
| CI: Confidence interval; Results given for brisk walking pace compared to slow pace. | | | |

# Cox model results by cardiometabolic disease status

|  | **No CMD** | **With CMD** |
| --- | --- | --- |
|  | Hazard ratio (95% CI) | Hazard ratio (95% CI) |
| Colon (proximal) | 1.06 (0.80-1.41) | 0.48 (0.30-0.77) |
| Small intestine | 0.35 (0.17-0.73) | 1.35 (0.48-3.86) |
| CI: Confidence interval; CMD: cardiometabolic disease.  Results given for brisk walking pace compared to slow pace. | | |

# Cox model results for never smokers

| **Cancer type** | **HR (95% CI)** | | **Cases** |
| --- | --- | --- | --- |
|  | Steady vs. slow pace | Brisk vs. slow pace |  |
| Anus | 1.11 (0.37-3.28) | 0.29 (0.08-1.06) | 50 |
| Liver (HCC) | 0.36 (0.18-0.72) | 0.22 (0.08-0.56) | 52 |
| Small intestine | 0.81 (0.35-1.90) | 0.50 (0.19-1.31) | 76 |
| Thyroid | 0.72 (0.38-1.36) | 0.50 (0.25-1.02) | 130 |
| Lung | 0.79 (0.50-1.26) | 0.73 (0.45-1.20) | 301 |
| Stomach (non-cardia) | 2.00 (0.45-8.85) | 3.66 (0.79-16.85) | 56 |

# Lung cancer risk adjusted for pack years of smoking

| **HR (95% CI)** | | **Cases** |
| --- | --- | --- |
| Steady vs. slow pace | Brisk vs. slow pace |  |
| 0.75 (0.65-0.87) | 0.61 (0.51-0.72) | 1544 |
| HR: Hazard ratio; CI: Confidence interval; Note: Pack years was defined as the daily number of cigarettes smoked, divided by twenty, and multiplied by the number of years of smoking | | |

# Negative control outcome

| **HR (95% CI)** | | **Cases** |
| --- | --- | --- |
| Steady vs. slow pace | Brisk vs. slow pace |  |
| 1.52 (0.96-2.42) | 1.05 (0.80-1.40) | 237 |
| HR: Hazard ratio; CI: Confidence interval; Note: The negative control outcome was defined as death by intentional self-harm obtained from death certificates (ICD-10 codes X60-X84). | | |

# P-values after correction for multiple comparisons

|  | **Average vs. Slow** | | **Brisk vs. Slow** | |
| --- | --- | --- | --- | --- |
| **Cancer** | Main p-value | False discovery rate | Main p-value | False discovery rate |
| Anus | 0.1388 | 0.5945 | **0.0015** | **0.0140** |
| Bladder | 0.1932 | 0.5945 | 0.3002 | 0.5680 |
| Brain, eye, CNS | 0.9875 | 0.9875 | 0.8761 | 0.9085 |
| Breast | 0.3255 | 0.5945 | 0.1038 | 0.3046 |
| Colon (distal) | 0.2479 | 0.5945 | 0.6650 | 0.7952 |
| Colon (proximal) | 0.5391 | 0.7547 | 0.4410 | 0.6813 |
| Corpus uteri | 0.8670 | 0.9635 | 0.3043 | 0.5680 |
| Gallbladder | 0.9423 | 0.9772 | 0.9393 | 0.9393 |
| Kidney | 0.2256 | 0.5945 | 0.4623 | 0.6813 |
| Larynx | 0.4791 | 0.7453 | 0.2858 | 0.5680 |
| Leukemia | 0.2585 | 0.5945 | 0.4194 | 0.6813 |
| Lips, oral cavity, pharynx | 0.2361 | 0.5945 | 0.0682 | 0.2728 |
| Liver (HCC) | 0.1345 | 0.5945 | **0.0005** | **0.0070** |
| Liver (IBDC) | 0.8209 | 0.9577 | 0.6083 | 0.7845 |
| Lung | **<0.0001** | **0.0003** | **<0.0001** | **0.0003** |
| Malignant melanoma | 0.7833 | 0.9552 | 0.5678 | 0.7845 |
| Multiple myeloma | 0.5319 | 0.7547 | 0.7701 | 0.8625 |
| Non-Hodgkin lymphoma | 0.4013 | 0.6610 | 0.3991 | 0.6813 |
| Oesophagus | 0.8947 | 0.9635 | 0.8750 | 0.9085 |
| Ovary | 0.2281 | 0.5945 | 0.1088 | 0.3046 |
| Pancreas | 0.2878 | 0.5945 | 0.2351 | 0.5680 |
| Prostate | **0.0479** | 0.5945 | 0.0943 | 0.3046 |
| Rectum | 0.6668 | 0.8891 | 0.6816 | 0.7952 |
| Renal pelvis, ureter | 0.7846 | 0.9552 | 0.6164 | 0.7845 |
| Small intestine | 0.3251 | 0.5945 | **0.0174** | 0.0845 |
| Stomach (cardia) | 0.3397 | 0.5945 | 0.2632 | 0.5680 |
| Stomach (non-cardia) | 0.1491 | 0.5945 | **0.0181** | 0.0845 |
| Thyroid | 0.2844 | 0.5945 | **0.0115** | 0.0805 |

## Flow chart of participant inclusion and exclusion

## Directed acyclic graph


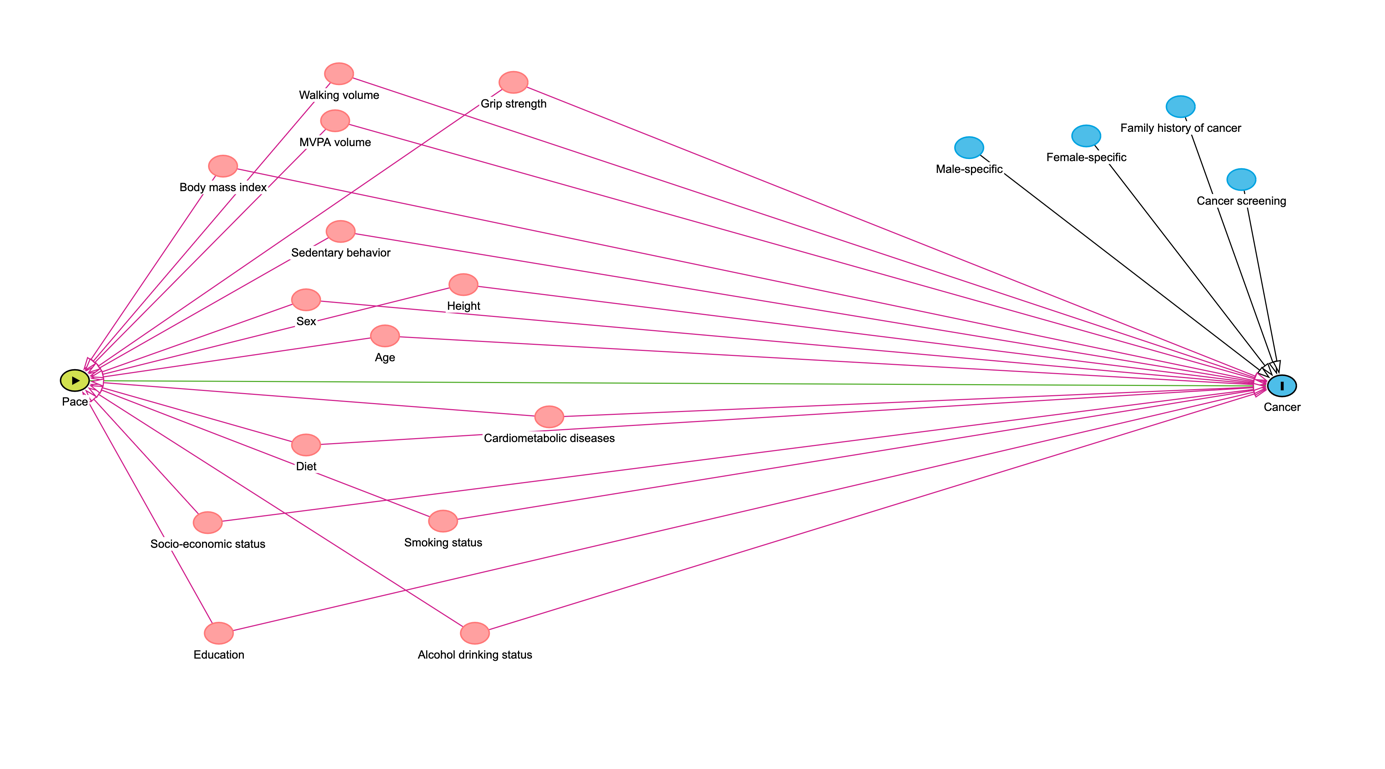


MVPA: Moderate-to-vigorous physical activity

Red: Ancestor of exposure and outcome, i.e., a confounder. Blue ancestor of outcome; these are only causes of the outcome, hence, not confounding variables, but adjusting for such variables tends to increase the power of statistical tests [1].

[1] VanderWeele TJ, Shpitser I. A New Criterion for Confounder Selection. Biometrics;2011;67(4):1406-13. https://doi.org/10.1111/j.1541-0420.2011.01619.x

##
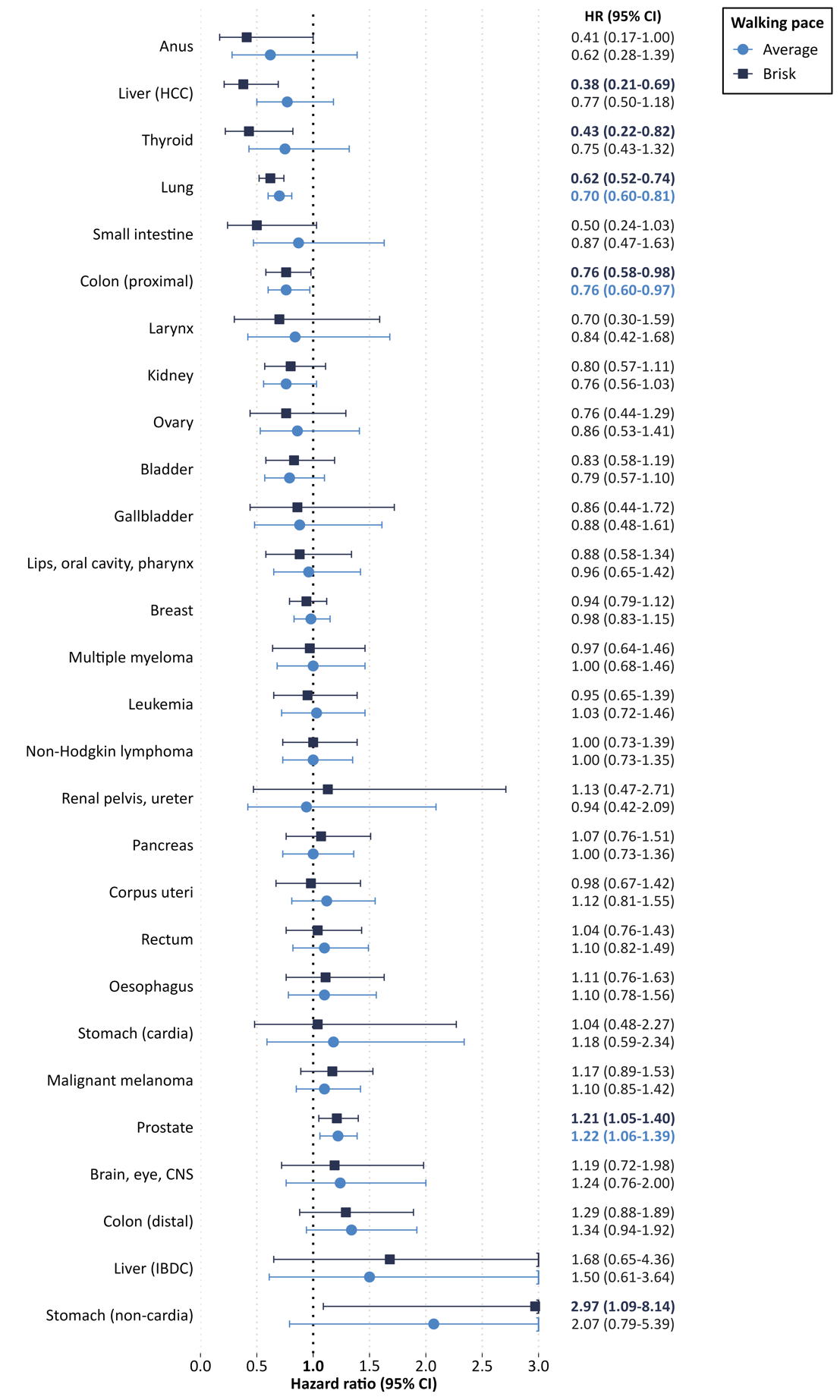
Cox model results after exclusion of first five years of follow-up

HCC: Hepatocellular carcinoma; IBDC: Intrahepatic bile duct cancer; CNS: Central nervous system. Note: Proportional hazards assumption did not hold for rectal cancer.

##
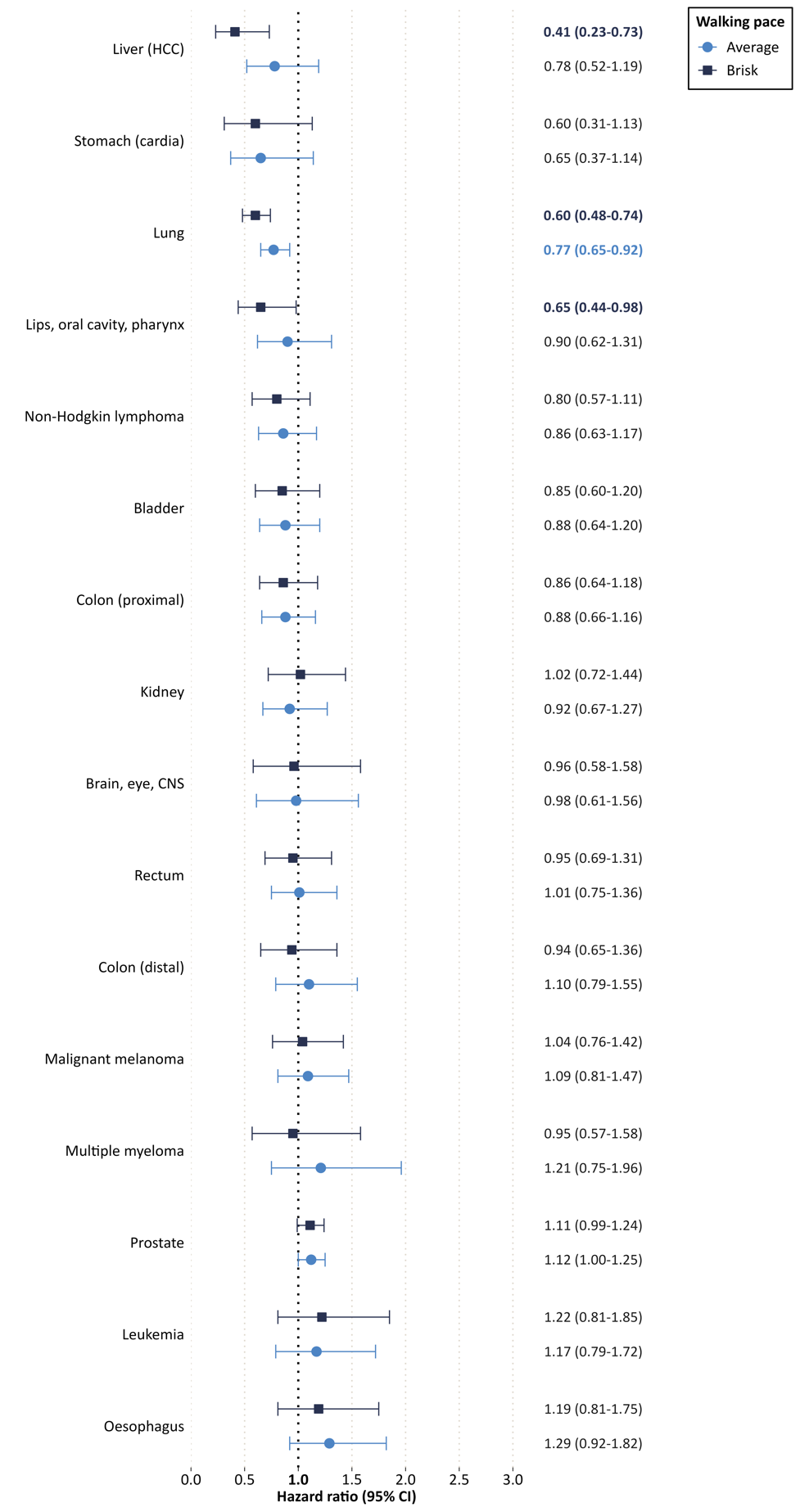
Cancer risk among men

Note: Proportional hazards assumption did not hold for rectal cancer.

##
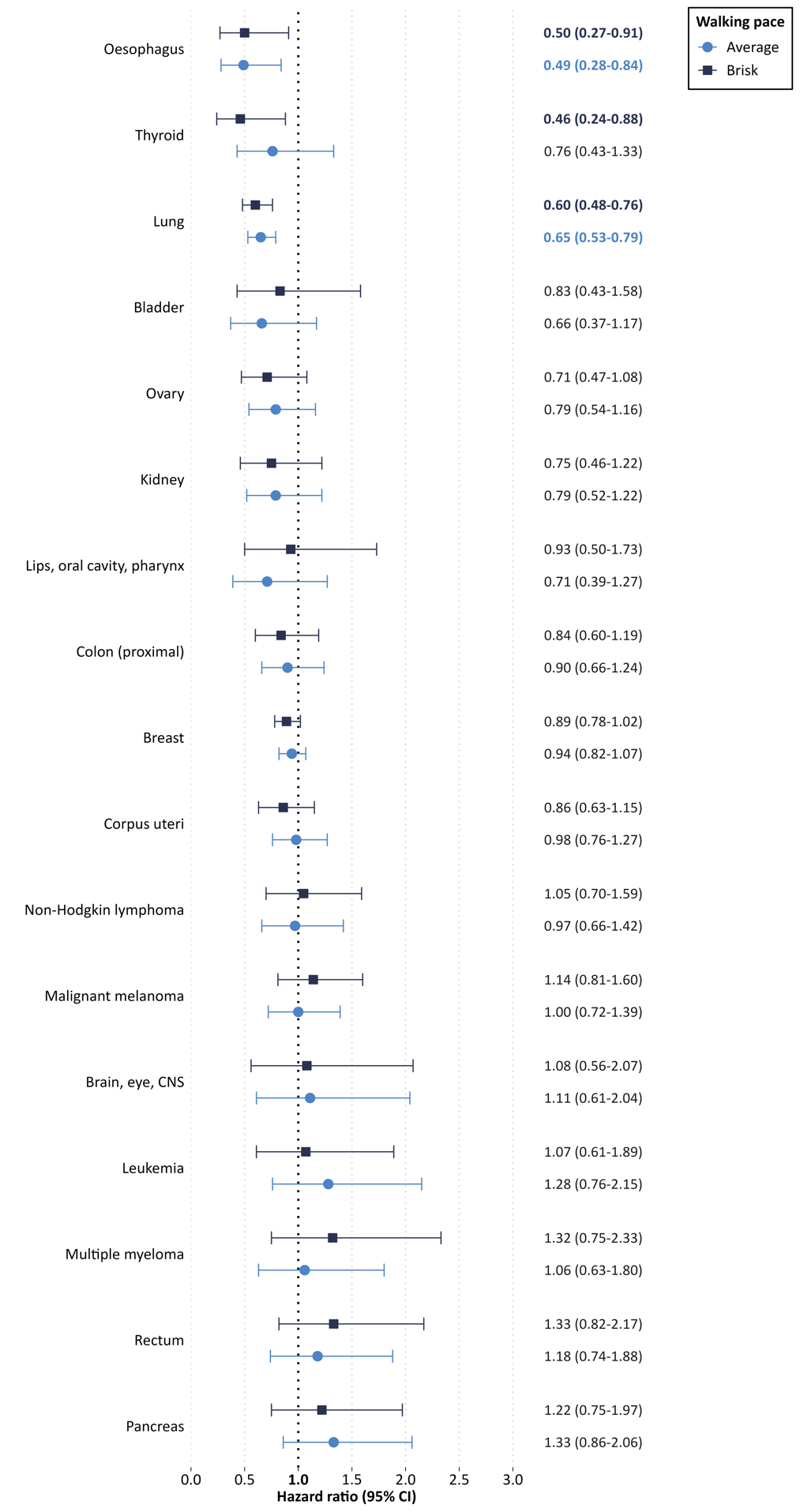
Cancer risk among women

Note: Proportional hazards assumption did not hold for rectal cancer.

## Cancer risk by age groups


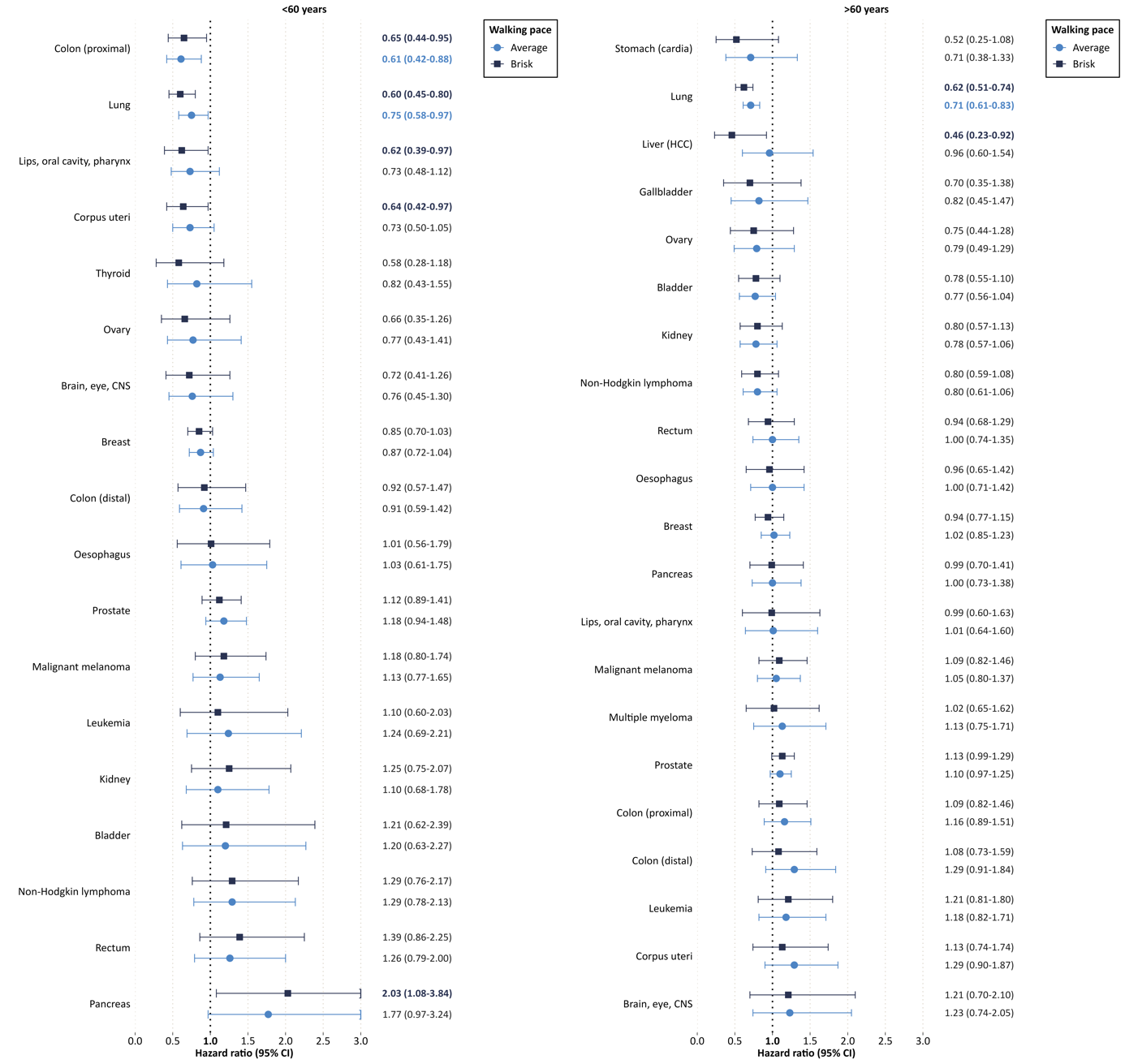


## Cancer risk after adjustment for overall health status


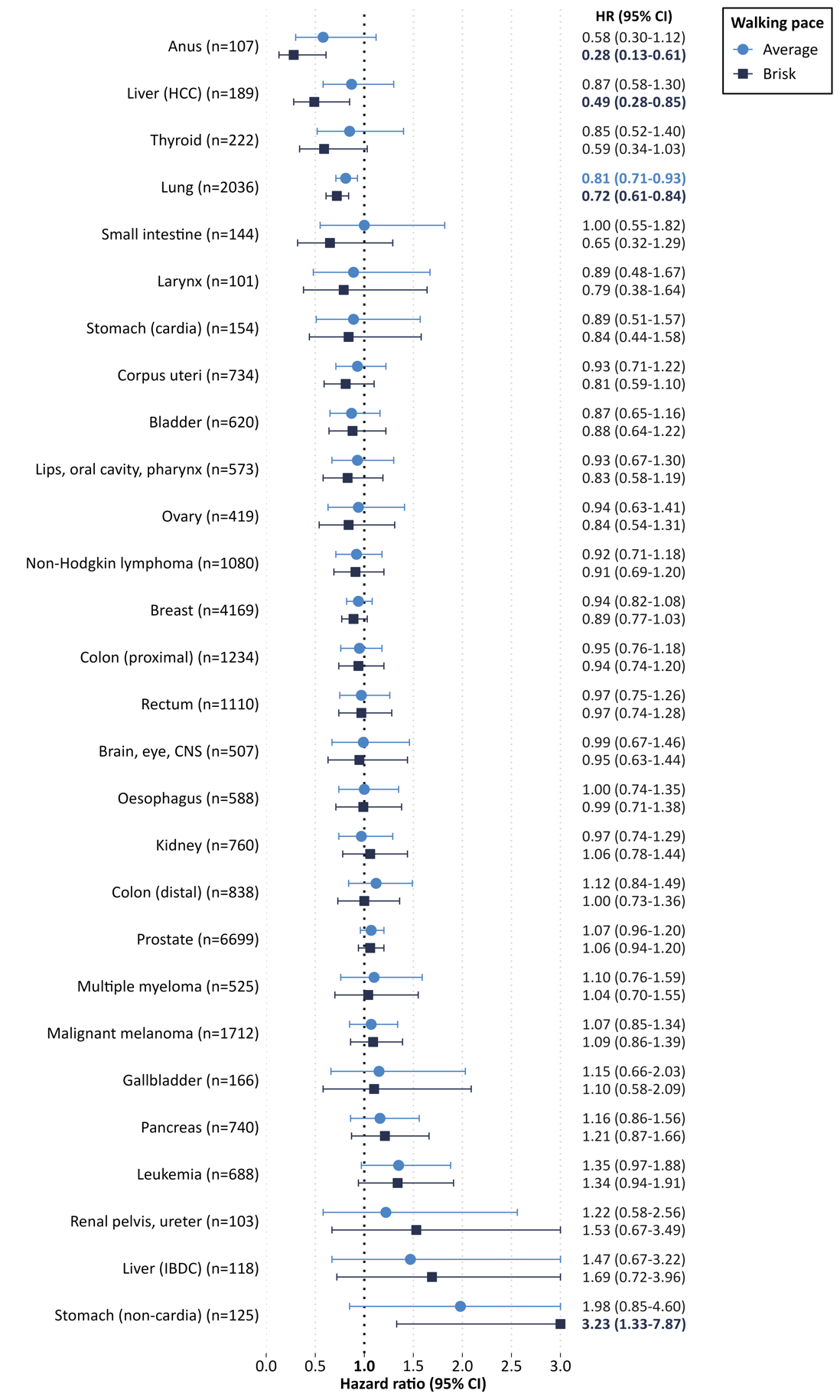


##
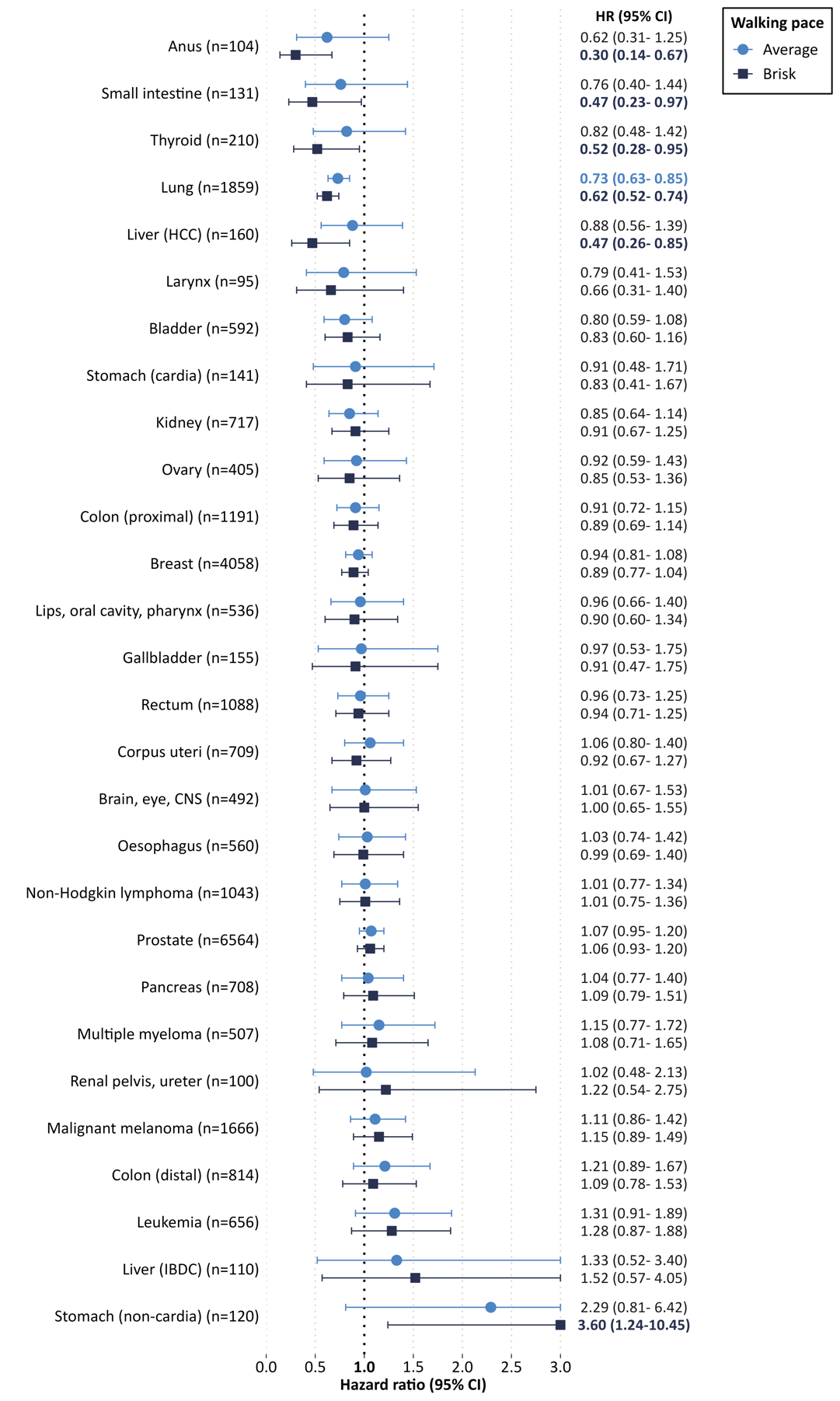
Cancer risk after exclusion of participants with poor self-rated health

## Cancer risk without adjustment for moderate-to-vigorous physical activity, walking volume, grip strength, and sedentary behavior


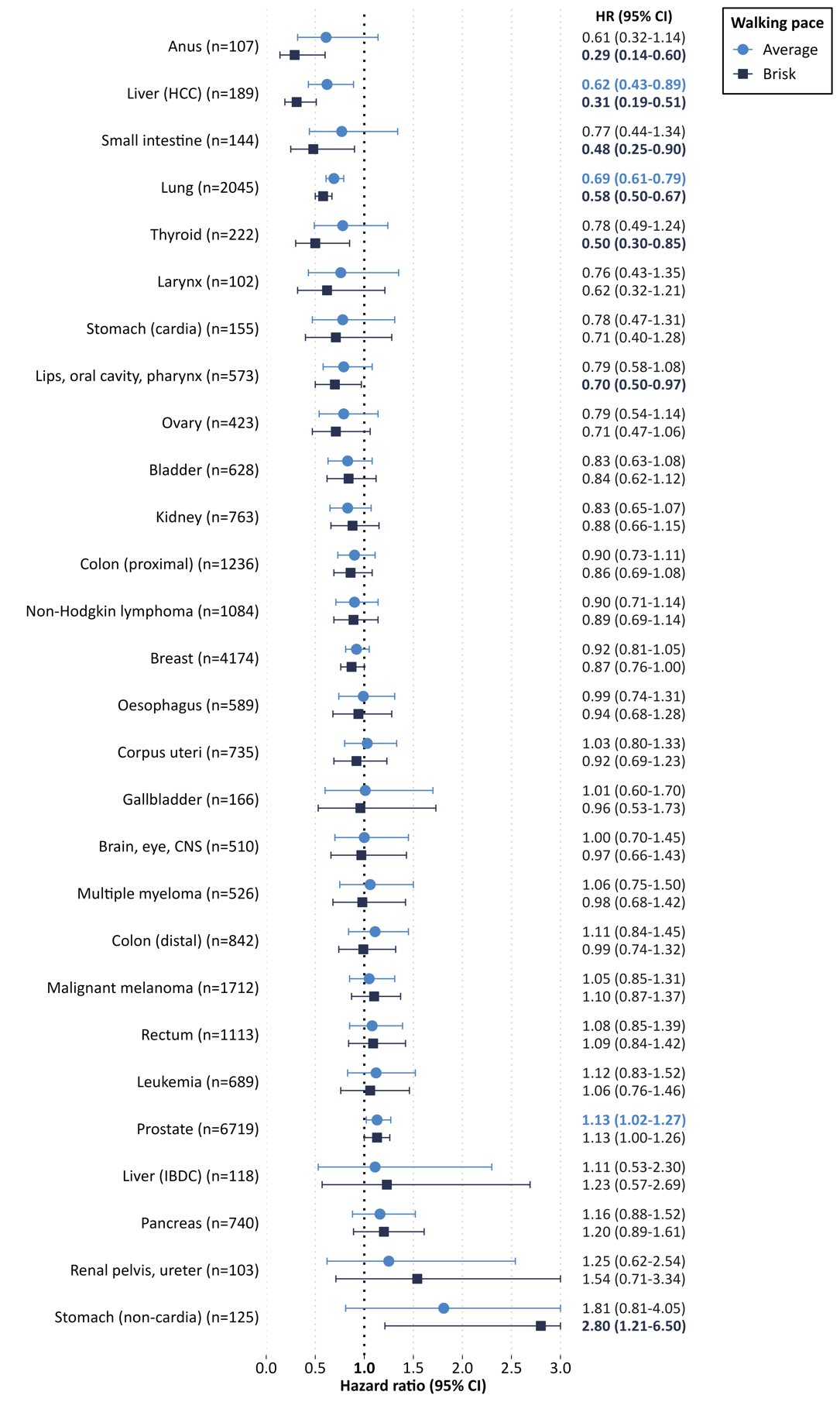

Supplement: Supplementary file 1 — Supplementary Material 1 [file 12885_2025_14258_MOESM1_ESM.docx]
